# Draft Genome Sequence of the Pathogenic Fungus *Scedosporium apiospermum*

**DOI:** 10.1128/genomeA.00988-14

**Published:** 2014-10-02

**Authors:** Patrick Vandeputte, Sarah Ghamrawi, Mathias Rechenmann, Agnès Iltis, Sandrine Giraud, Maxime Fleury, Christopher Thornton, Laurence Delhaès, Wieland Meyer, Nicolas Papon, Jean-Philippe Bouchara

**Affiliations:** a L’UNAM Université, Université d’Angers, Groupe d’Etude des Interactions Hôte-Pathogène, Angers, France; b Laboratoire de Parasitologie-Mycologie, Centre Hospitalier Universitaire, Angers, France; c Genostar, Montbonnot, France; d Hybridoma, Exeter, United Kingdom; e Université de Lille II, Biologie et Diversité des Pathogènes Eucaryotes Emergents, Lille, France; f Molecular Mycology Research Laboratory, Centre for Infectious Diseases and Microbiology, Sydney Medical School, Westmead Hospital, Marie Bashir Institute for Infectious Diseases and Biosecurity, University of Sydney, Westmead Millenium Institute for Medical Research, Westmead, New South Wales, Australia; g Université de Tours, Biomolécules et Biotechnologie Végétales, Faculté de Pharmacie, Tours, France

## Abstract

The first genome of one species of the *Scedosporium apiospermum* complex, responsible for localized to severe disseminated infections according to the immune status of the host, will contribute to a better understanding of the pathogenicity of these fungi and also to the discovery of the mechanisms underlying their low susceptibility to current antifungals.

## GENOME ANNOUNCEMENT

*Scedosporium apiospermum* is a soil-borne opportunistic pathogen responsible for cutaneous or subcutaneous mycetomas following traumatic inoculation of fungal elements and for respiratory tract infections. Moreover, it becomes a redoubtable pathogen in immunocompromised patients, where it may cause disseminated infections in the skin, eye, bones, joints, and deep organs, such as the heart and central nervous system (1). Species of the *Scedosporium apiospermum* complex are the second most frequent molds, after *Aspergillus fumigatus*, and colonize the respiratory tract of cystic fibrosis patients, causing in this clinical context respiratory infections such as bronchitis and allergic broncho-pulmonary mycoses, or disseminated life-threatening infections in cases of immunodeficiency such as corticosteroid-induced diabetes or after lung transplantation. Here we report the sequencing and annotation of the genome of one of the major species within this closely related species complex.

The genome sequence of *Scedosporium apisopermum* strain IHEM 14462, isolated in 1998 from a sputum sample from a cystic fibrosis patient in Tours, France, was resolved by two distinct high-throughput Illumina sequencing technologies on an HiSeq2000: a paired-end run, sequencing on average 50 bp at each extremity of approximately 120.3 million 250-bp inserts, and a mate-pairs run, sequencing on average 50 bp at each extremity of approximately 82.2 million 4-kb inserts. *De novo* assembly was achieved by an additional single-molecule real-time sequencing on a PacBio RSII instrument (Pacific Biosciences), thus fully sequencing 273,000 inserts with a mean size of 2.3 kb. After trimming bad-quality Illumina runs, the sequences were assembled by Genostar (Montbonnot, France) in 3,744 contigs with the CLC Genomics Workbench version 6.0.2 (http://www.clcbio.com/products/clc-genomics-workbench). PacBio RSII reads were then used to generate scaffolds from the contigs using the softwares BLASR (2) and SSPACE Premium scaffolder version 2.3 (3). Subsequently, gaps were closed with GapFiller version 1.10 (4). Finally, 176 scaffolds were obtained with a mean size of 246,804 bp, representing a total length of 43.4 Mbp.

The genome annotation was performed by Genostar; prediction of coding DNA sequences (CDSs) was performed with Augustus version 2.5.5 (5), trained on an algorithm optimized for *Neurospora crassa*. The 10,919 putative CDSs identified were annotated by BLASTP (6) against the TrEmbl database (7). A function was inferred for a given CDS when the deduced protein sequence shared at least 80% similarity and 40% identity with a protein of known function in the TrEmbl database. A putative function was attributed to the remaining CDSs by functional domain searches through the Pfam database (8). Thus, a function was assigned to 8,818 out of the 10,919 CDSs (80.75%). For 813 CDSs, the functional annotation was refined by attribution of the enzyme classification numbers.

Genomic data will greatly improve our comprehension of the pathogenic mechanisms underlying scedosporiosis and will help our understanding of the low susceptibility of *Scedosporium apiospermum* to currently available antifungal drugs (9).

### Nucleotide sequence accession numbers.

This whole-genome shotgun project has been deposited at DDBJ/EMBL/GenBank under the accession number JOWA00000000. The version described in this paper is the first version, JOWA01000000.
